# Nutritional Manipulation for the Primary Prevention of Gestational Diabetes Mellitus: A Meta-Analysis of Randomised Studies

**DOI:** 10.1371/journal.pone.0115526

**Published:** 2015-02-26

**Authors:** Ewelina Rogozińska, Monica Chamillard, Graham A. Hitman, Khalid S. Khan, Shakila Thangaratinam

**Affiliations:** 1 Women’s Health Research Unit, Centre of Primary Care and Public Health, Blizard Institute, Barts and the London School of Medicine and Dentistry, Queen Mary University of London, London, United Kingdom; 2 Multidisciplinary Evidence Synthesis Hub (mEsh), Centre of Primary Care and Public Health, Blizard Institute, Barts and the London School of Medicine and Dentistry, London, United Kingdom; 3 Centro Rosarino Estudios Perinatales, Rosario, Santa Fe, Argentina; 4 Centre of Diabetes, Blizard Institute, Barts and the London School of Medicine and Dentistry, Queen Mary University of London, London, United Kingdom; INIA, SPAIN

## Abstract

**Introduction:**

The rise in gestational diabetes (GDM), defined as first onset or diagnosis of diabetes in pregnancy, is a global problem. GDM is often associated with unhealthy diet and is a major contributor to adverse outcomes maternal and fetal outcomes. Manipulation of nutrition has the potential to prevent GDM.

**Methods:**

We assessed the effects of nutritional manipulation in pregnancy on GDM and relevant maternal and fetal outcomes by a systematic review of the literature. We searched MEDLINE, EMBASE, and Cochrane Database from inception to March 2014 without any language restrictions. Randomised controlled trials (RCT) of nutritional manipulation to prevent GDM were included. We summarised dichotomous data as relative risk (RR) and continuous data as standardised mean difference (SMD) with 95% confidence interval (CI).

**Results:**

From 1761 citations, 20 RCTs (6,444 women) met the inclusion criteria. We identified the following interventions: diet-based (n = 6), mixed approach (diet and lifestyle) interventions (n = 13), and nutritional supplements (*myo*-inositol n = 1, diet with probiotics n = 1). Diet based interventions reduced the risk of GDM by 33% (RR 0.67; 95% CI 0.39, 1.15). Mixed approach interventions based on diet and lifestyle had no effect on GDM (RR 0.95; 95% CI 0.89, 1.22). Nutritional supplements probiotics combined with diet (RR 0.40; 95% CI 0.20, 0.78) and *myo*-inositol (RR 0.40; 95% CI 0.16, 0.99) were assessed in one trial each and showed a beneficial effect. We observed a significant interaction between the groups based on BMI for diet-based intervention. The risk of GDM was reduced in obese and overweight pregnant women for GDM (RR 0.40, 95% CI 0.18, 0.86).

**Conclusions:**

Nutritional manipulation in pregnancy based on diet or mixed approach do not appear to reduce the risk of GDM. Nutritional supplements show potential as agents for primary prevention of GDM.

## Introduction

Gestational diabetes mellitus (GDM), defined as carbohydrate intolerance first diagnosed in pregnancy, is on the rise worldwide. [1] An increase in the number of mothers entering pregnancy as obese and with advancing maternal age has contributed to this escalation in rates of GDM. Women with GDM and their children are at risk of adverse outcomes in pregnancy and in the long term. [2] About half of mothers with GDM are expected to develop Type 2 diabetes within five years after pregnancy. [3] In the offspring it is a major contributor to obesity and Type 2 diabetes in later life. [4] There is a need for safe, simple, effective and acceptable interventions that prevent the development of GDM. Such an approach has the potential to improve maternal and child health, with significant savings to the health care system.[5] 

Interventions that prevent Type 2 diabetes might reduce the risk of GDM. Nutritional manipulation based on diet and lifestyle is known to significantly lower the rates of Type 2 diabetes in non-pregnant individuals. [6] This beneficial effect could be attributed either to the reduction in the calorie intake, or to the effect of individual components of diet such as yoghurt and cereals that are rich in probiotics, fibre and vitamins. [7,8] Currently no such interventions are offered to mothers as part of routine antenatal care to reduce GDM. Systematic reviews to-date, are based on limited number of studies, and have not provided conclusive evidence on the benefits of nutritional interventions in preventing GDM. [9–11] Furthermore, individual studies are underpowered to reliably estimate reductions in the rates of GDM. [12] 

There is a need to collate the accruing evidence on nutritional manipulation. We systematically reviewed the effectiveness of nutritional manipulation in pregnancy with mainly diet-based interventions; mixed approach with diet and lifestyle; and nutritional supplements in preventing GDM.

## Methods

We undertook the systematic review with a prospective protocol in line with current recommendations [13] and reported according to the PRISMA guidelines. [14] 

This project is secondary research only so requires no ethical approval.

### Literature search and study selection

A comprehensive search of the relevant literature was performed in electronic databases (MEDLINE, EMBASE and the Cochrane Library) from inception to March 2014. The search strategy was designed by combining the search terms: “diet,” “vitamins,” “probiotics,” “gestational,” “diabetes” and “pregnancy” using their word variants and Boolean operators AND and OR as appropriate. No language restrictions were applied. We contacted the authors of primary studies to obtain any relevant unpublished data. Additionally, we searched the reference lists of the included studies for relevant literature.

Studies were selected in a two-stage process. We screened the titles and abstracts against the pre-specified inclusion criteria for relevant citations. This was followed by assessment of the full texts of the selected abstracts. We included randomised studies that evaluated the effect of nutritional interventions in pregnancy: diet based advice, mixed approach (combination of diet and lifestyle including physical activity) and nutritional supplements that have the potential to reduce the risk of GDM such as vitamin-D, *myo*-inositol and probiotics. We did not include studies that evaluated only physical activity. The comparator was standard antenatal care. Primary prevention of GDM was the outcome of interest. The secondary outcomes were maternal and fetal complications such as pre-eclampsia, mode of delivery, gestation at delivery, birth weight of the fetus, neonatal death and neonatal intensive care unit admissions. We included studies on low risk and high-risk women. Women were classified as high risk if they have at least one of the following characteristics: obesity, previous history of GDM or fetal macrosomia, advanced maternal age, and family history of diabetes. [15] Two independent reviewers (ER and MC) selected the studies. Any discrepancy between them was resolved by a third reviewer (ST).

### Study quality assessment and data extraction

Two independent reviewers (ER and MC) assessed the study quality and extracted the data using pre-designed forms. We assessed the risk of bias [16] in the following domains: sequence generation, allocation concealment, blinding, incomplete outcome data and selective reporting. Data were extracted in 2x2 tables for dichotomous outcomes. For continuous outcomes we extracted data on mean and standard deviation for both the groups. When more than one definition was used for GDM in a study, we extracted data for outcomes using the most recent diagnostic criteria. Any discrepancies were resolved by discussion with the third reviewer (ST).

### Data synthesis

Data were summarised as risk ratio (RR) with 95% confidence interval (CI) for dichotomous outcomes, and standardised or weighted mean difference with 95% CI for continuous outcomes using the random effects model. We assessed statistical heterogeneity between trials by using the I^2^ statistic. We undertook subgroup analysis planned *a priori* to explore whether the effect on the outcome would vary according to the type of intervention, and Body Mass Index (BMI), and risk status of the participants for GDM. The subgroup difference was evaluated using Chi squared test. When more than one intervention was compared to standard care in a study, we chose the combined intervention over the individual diet or nutritional supplement for the pooled analysis. We used random effects model for meta-analysis. Sensitivity analysis was undertaken by substituting the individual intervention instead of the combined method to assess for any change in the summary estimates of effects. We used Harbord’s modified test to assess for publication bias [17] and potential small study effect. All analyses were performed with Review Manager (RevMan version 5.2) and Stata software (version 11).

## Results

### Study selection

**Fig 1:**
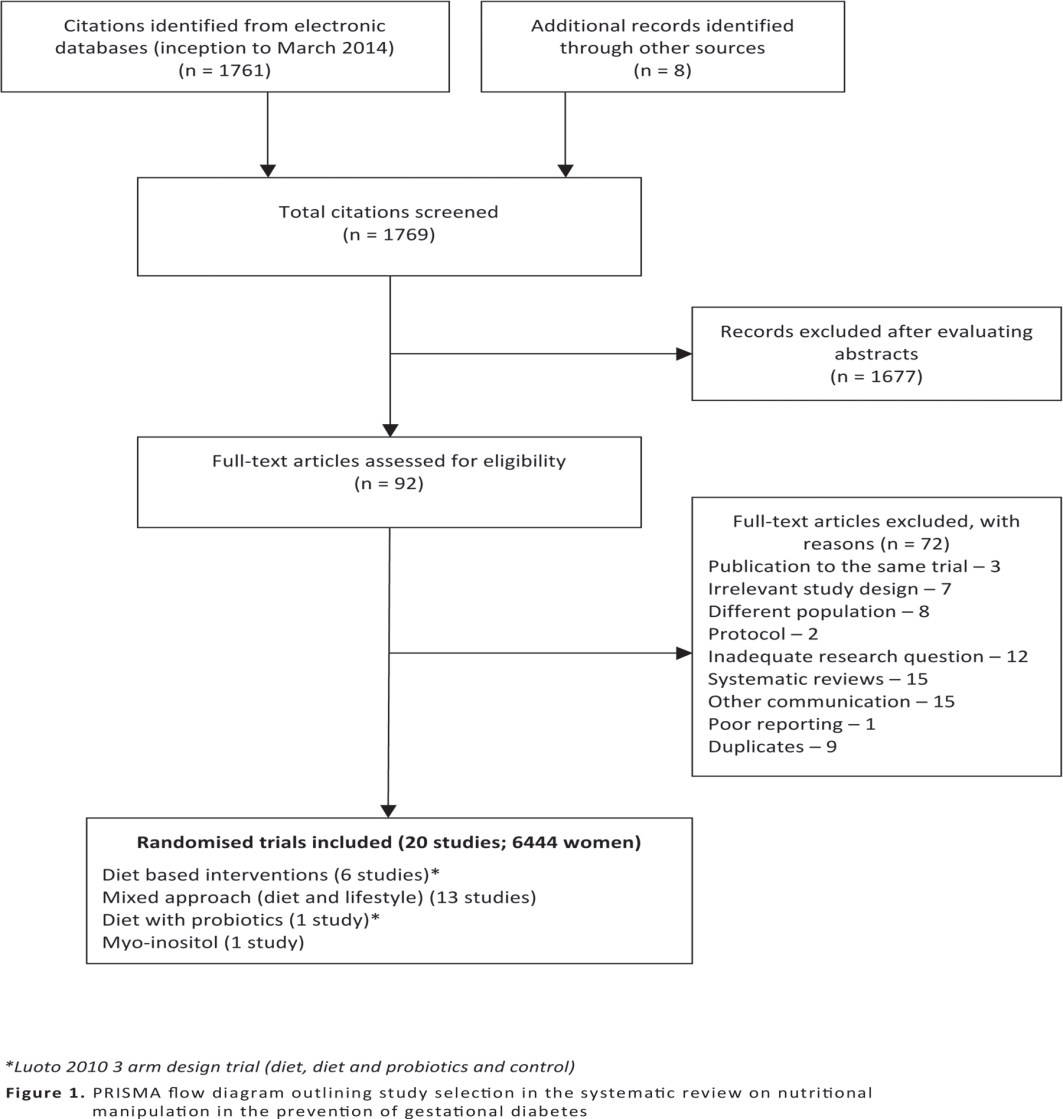
PRISMA flow diagram outlining study selection in the systematic review on nutritional manipulation in the prevention of gestational diabetes.

Our initial search in electronic databases yielded 1761 citations. Further eight studies were identified from the reference lists of the selected studies. Twenty RCTs with 6,444 women were included in the review. [18–37] The process of study identification and study selection is provided in Fig. 1.

### Characteristics of the included studies

The following nutrition based methods were evaluated: diet based interventions (5 RCTs; 1,309 women) [25,31,33,36,37], mixed (diet and lifestyle) approach (13 RCTs; 4,745 women) [18,20–24,27–30,32,34,35] and nutritional supplements: *myo*-inositol (1 RCT; 220 women) [19] and diet with probiotics (1 RCT; 170 women) [26] .

Of the 20 included studies, 13 RCTs were on women at risk of developing gestational diabetes [18,19,21,22,25,30–37] and seven included any risk women [20,23,24,26–29]. Twelve RCTs examined the effect of the intervention in either obese or/and overweight women [18,20,22,27,30–35,37] and eight included women of any BMI. [19,23–26,28,29,36] 

The interventions varied in their composition, especially those based on diet. The diet based strategy included low glycaemic index diet [36] , and restricted energy intake according to the individual requirements. [37] The mixed approach group delivered a combination of diet and lifestyle including physical activity. The nutritional supplement *myo*-inositol was provided as a 2 g dose twice a day with 200 ug folic acid. The probiotics Lactobacillus Rhamnosus GG and Bifidobacterium lactis Bb12 in dose of 10^10^ colony forming units were taken every day in addition to intensive dietary counselling. [26] 

The interventions were delivered in groups or as a one-to-one contact session, and often in more than one step. The nutritional advice was accompanied by psychological input in some studies. [23,31] Participants were provided with food diaries to record their food intake and the dieticians tailored the intervention according to the caloric requirements. Most of the interventions from the mixed approach category were based on the national recommendations on healthy eating in pregnancy, with additional input provided by trained healthcare personnel. The mixed approach category aggregates studies with complex interventions targeting weight gain from different angles; ranging from change in a type of consumed foods and daily physical activity pattern [23,27] , behavioural change [18,22] to weight gain monitoring only [24] . All the interventions were commenced before 28 weeks at varied time points in the first or second trimester.

The definition of GDM varied between the studies (Table 1). The following maternal outcomes were evaluated: preterm delivery, caesarean section, induction of labour, pre-eclampsia and pregnancy induced hypertension. The fetal outcomes included birth weight, shoulder dystocia and admission to neonatal intensive care unit (NICU). The follow up period varied from 6 weeks after delivery to the end of exclusive breastfeeding.

**Table 1:** Clinical characteristics of included studies evaluating the effectiveness of nutrition manipulation in primary prevention of gestational diabetes mellitus (GDM).

Study, Year	Number of patient	Methods	Participants	Intervention	Control	Outcomes
**Luoto 2010** ^**26**^	**256**	**Methods of Randomization:** Random assignment to one of three groups according to computer-generation block of six women	**Inclusion Criteria:** Women in their early pregnancy with no chronic metabolic diseases	**Group 1:** Intensive dietary counselling	Dietary counselling according to a national programme and placebo capsules	**GDM** (Definition: Fourth International Workshop-Conference on GDM ≥ 4·8 mmol/l at baseline, or ≥10·0 mmol/l at 1 h, ≥8·7 mmol/l at 2 h.)
		**Allocation concealment:** Sealed envelopes with subject number	**Exclusion Criteria:** Not specified	**Group 2**: Probiotic capsules Lactobacillus, Rhamnosus GG and Bifidobacterium lactis Bb12 at a dose of 10^10^ colony forming units day each before 17 weeks of gestation once a day		caesarean section, birth weight, gestational age at delivery
		**Blinding:** Double blind manner. All personnel involved in handling or analyzing blood samples was blinded to the intervention				
**D’Anna 2013** ^**19**^	**220**	**Methods of Randomization:** A computer randomization with 1:1 allocation	**Inclusion Criteria:** Caucasian women with singleton pregnancy without GDM whose first degree relative was affected by type-2 diabetes; whose BMI<30kg/m^2^; fasting plasma glucose <126mg/dl and random glycaemia <200mg/dl.	2g myo-inositol plus 200ug acid folic twice a day since 12–13 weeks of gestation	200ug acid folic twice a day since 12–13 weeks of gestation.	**GDM** (Definition: ADIPS criteria—75 g, 2 hours glucose tolerance test, with cutoff values of >92 mg/dl for time 0, >180 mg/dl after 1 hour, >153 mg/dl after 2 hours. At least one of three values over or equal to the cutoff was enough for diagnosis of GDM)
		**Allocation concealment:** Not reported	**Exclusion Criteria:** Pre-pregnancy BMI>30kg/m^2^, previous GDM, pre-gestational diabetes, first trimester glycosuria, first degree relative not affected by type2 diabetes, fasting plasma glucose >126mg/dl or random glycaemia >200mg/dl, Twin pregnancy, any therapy using corticoids, Not Caucasian or with PCOS			gestational hypertension, preterm delivery, caesarean section,
		**Blinding:** Open Label Trial				macrosomia, respiratory distress syndrome, shoulder dystocia, neonatal hypoglycemia.
**Korpi-Hyovalti 2012** ^**25**^	**54**	**Methods of Randomization:** A computed randomization	**Inclusion Criteria:** Meeting one of the following criteria: BMI >25, previous history of GDM, previous macrosomia (>4,500g),>40 years, family history of diabetes, venous plasma glucose concentration after 12 h overnight fasting was 4,8 to 5,5mmol/l 2h oral glucose tolerance test plasma <7,8	Diet: rich in vegetables, berries and fruits, fat-free and low-fat dairy products, low-fat meat, soft margarines and vegetable oils and whole-grain products. Recommended energy intake: normal weight women—126 kJ/kg per day; overweight women—105 kJ/kg per day. The goal of in pregnancy weight gain: 12·5–18 kg for underweight women, 11·5–16·0 kg for normal-weight women and 7–11·5 kg for overweight women.	General information on diet and physical activity in a single session to decrease the risk of GDM	**GDM** (Definition: Not reported)
		**Allocation Concealment:** Not reported				
		**Blinding:** Open Label Trial	**Exclusion Criteria:** Diagnosed GDM at inclusion			birth weight, gestational age at delivery
**Quinvilan 2011** ^**31**^	**132**	**Methods of Randomization:** A computer randomization.	**Inclusion Criteria:** Pregnant women carrying fetus without any known anomalies; able to attend hospital for antenatal care; underweight or obese according to standard BMI ranges	Four step multidisciplinary antenatal care: continuity of care provider, weighing on arrival, brief dietary intervention by food technologist at every antenatal visit and psychological assessment and intervention if indicated	Routine public antenatal care	**GDM** (Definition: Women underwent on consecutive days, a 75g fasting 2-hours glucose tolerance test and then 100g fasting 3-hours glucose tolerance. > 6.6 mmol/l decreased gestational glucose tolerance >7.7 mmol/l GDM)
		**Allocation concealment:** Sealed opaque envelopes				GWG
		**Blinding:** Outcomes data were audited by a nurse blinded to randomization status	**Exclusion Criteria:** Multiple gestations.			birth weight
**Thornton 2009** ^**33**^	**257**	**Methods of Randomization:** A random-number tables	**Inclusion Criteria:** Pregnant with a single fetus between 12–28 weeks of gestation. BMI≥30kg/m^2^	The nutritional programme with dietary guidelines similar to ones used for patients with GDM. All women were asked to record in a diary all the foods and beverages consumed during each day	Standard Care	**GDM** (Definition: Not reported)
		**Allocation concealment:** Sequentially numbered envelopes				GWG, pre-eclampsia, gestational hypertension, duration of pregnancy, induction of labour, caesarean Section
		**Blinding:** Not reported	**Exclusion Criteria:** Patient with pre-existence diabetes, hypertension or chronic renal disease			birth weight, macorosomia, Apgar
**Walsh 2012** ^**36**^	**800**	**Methods of Randomization:** A computer generated allocations in ratio 1:1	**Inclusion Criteria:** Women who had previously delivered a macrosomic baby (>4kg).	Health eating guidelines for pregnancy with focus on low glycaemic index	Routine Antenatal Control	**GDM** (Definition: Carpenter and Coustan /ADA)
		**Allocation concealment:** Sealed opaque envelopes	**Exclusion Criteria:** Women with any underlying medical disorder, including a previous history of gestational diabetes; gestation beyond 18 weeks and multiple pregnancy			Maternal glucose intolerance, duration of pregnancy, mode of delivery, anal sphincter injuries, weight gain in recommendation to IOM, postpartum haemorrhage,
		**Blinding:** According to the authors blinded randomized trial of diet intervention is not possible				birth weight, shoulder dystocia
**Wolff 2008** ^**37**^	**53**	**Methods of Randomization:** A computerized randomization	**Inclusion Criteria:** Pregnant obese women with BMI >30kg/m^2^, in their early pregnancy (<15weeks) of Caucasian origin	10 consultations 1 hour each with a trained dietitian during the pregnancy. A healthy diet according to the official Danish dietary recommendations. The energy intake was restricted based in individually estimated energy requirement and estimated energetic cost of fetal growth	No consultations with dietitian and no energy intake or gestational weight gain restrictions	**GDM** (Definition: Not reported)
		**Allocation concealment:** Not reported	**Exclusion Criteria:** Smoking, below 18 and above 45 years, with multiple pregnancy, known medical complication which could affect fetal growth adversely or contraindicate limitation to weight gain			fasting blood samples for measurements of serum insulin, serum leptin, and blood glucose, GWG, daily food intake, fasting bloods samples for measurement of insulin, leptin and blood glucose, prolonged pregnancy, cesarean section, pre-eclampsia, gestational hypertension,
		**Blinding:** The physicians and midwives were blinded in regard to the treatment assignment; the women were asked not to reveal the allocation by the randomization				birth weight, Apgar score, infant length at delivery, placental weight
**Bogaerts 2012** ^**18**^	**141**	**Methods of Randomization:** Randomization took place by choosing one opaque envelope containing a ticket indicating one of the three groups	**Inclusion Criteria:** Obese pregnant women (BMI >29kg/m^2^) attending the antenatal clinic before 15 weeks pregnancy were informed by their gynecologist or midwife about the study.	The four sessions were scheduled: The sessions focused on the relation between energy intake and energy expenditure based on the active and healthy food pyramid for pregnant women. Recommendations for a healthy and balanced diet were based on the official National Dietary Recommendations and consisted of 50–55% carbohydrate intake, 30–35% fat intake and 9–11% protein energy intake.	Routine antenatal care	**GDM** (Definition: ADIPS criteria)
		**Allocation Concealment:** Opaque envelopes	**Exclusion Criteria:** Gestational age >15 weeks, Pre-existing type 1 diabetes, multiple pregnancy, primary need for nutritional advice and insufficient knowledge of the Dutch language.			GWG, gestational age at delivery, gestational hypertension / pre-eclampsia, levels of state anxiety mood depression
		**Blinding:** Not reported				birth weight, induction of labour, caesarean section
**Dodd 2014** ^**20**^	**2212**	**Methods of Randomization:** The computer generated randomization schedule with balanced variable blocks in the ratio 1:1 prepared by third party	**Inclusion Criteria:** BMI ≥25kg/m^2^ and singleton pregnancy between 10 to 20 weeks’ gestation	Dietary advice consistent with current Australian standards (maintenance of balance between carbohydrates, fat, and protein; reduction in intake of foods high in refined carbohydrates and saturated fats; increase of fiber intake. Physical activity advice primarily aiming women to increase their amount of walking and incidental activity	Standard care according to state-wide perinatal practice and local hospital guidelines which did not covered routine provision of advice related to diet, exercise, or gestational weight gain	**GDM** (Definition: positive 75g oral glucose tolerance test result with fasting blood glucose ≥5.5 mmol/L or 2 hour ≥7.8 mmol/L)
		**Allocation Concealment:** Allocation revealed using telephone central randomization service	**Exclusion Criteria**: Women with type 1 or 2 diabetes diagnosed before pregnancy			GWG, cesarean section, preterm delivery, gestational hypertension,, induction of labour,
		**Blinding:** Outcome assessors were blinded to the treatment group allocated				shoulder dystocia, neonatal death, admission to NICU
**Guelinckx 2010** ^**21**^	**130**	**Methods of Randomization:** Random allocation by using block randomization	**Inclusion Criteria:** Obese (BMI >29 kg/m^2^) white women attending prenatal clinic before 15 weeks of gestation	Recommendation on balanced, healthy diet following official National Dietary Recommendations	Routine prenatal care	**GDM** (Definition: Carpenter and Coustan criteria)
		**Allocation Concealment:** Not reported.	**Exclusion Criteria**: Pre-existing diabetes or developing GDM, multiple pregnancy, recruitment after 15 weeks of gestational age, premature labor (<37 weeks of gestation), primary need for nutritional advice in case of a metabolic disorder, kidney problems, Crohn disease, and any allergic conditions.			GWG, gestational age at delivery, gestational hypertension, pre-eclampsia, induction of labour, caesarean section,
		**Blinding:** Not reported.				birth weight
**Harrison 2011** ^**22**^	**228**	**Methods of Randomization:** Computer-generated randomized sequencing	**Inclusion Criteria:**12 to 15 weeks gestation, overweight (BMI ≥ 25 or ≥ 23kg/m^2^ if high-risk ethnicity [Polynesia, Asia, and Africa populations]) or obese (BMI ≥ 30 kg/m^2^), and at increased risk for developing GDM identified by a validated risk prediction tool	Four-session behavior change lifestyle intervention based on the Social Cognitive Theory (adapted from HeLP-her program) The sessions provided comprised of information of pregnancy-specific dietary advice, simple healthy eating and physical activity messages and simple behavioral change strategies.	A brief, single education session based on Australian Dietary and Physical Activity Guidelines.	**GDM** (Definition: ADIPS criteria including the presence of either a fasting venous plasma glucose level of ≥99 mg/dl (≥5.5 mmol/l) and/or a 2-h level of ≥144 mg/dl (≥8.0 mmol/l); and International Association of Diabetes and Pregnancy Study Group (IADPSG) criteria—fasting venous plasma glucose level of ≥91.8 mg/dl (≥5.1 mmol/l), a 1-h plasma glucose of ≥180 mg/dl (≥10.0 mmol/l), or a 2-h glucose level of ≥153 mg/dl (≥8.5 mmol/l)
		**Allocation Concealment:** Sealed opaque envelopes	**Exclusion Criteria:** Multiple pregnancies, diagnosed type 1 or 2 diabetes, BMI ≥ 45 kg/m2, and pre-existing chronic medical conditions			GWG, physical activity, risk perception
		**Blinding:** Care providers, investigators and outcome data analyzers were blinded to group allocation				
**Hui 2011** ^**23**^	**224**	**Methods of Randomization:** A computer-generated randomization table	**Inclusion Criteria:** Pregnant women (<26 weeks of pregnancy) with no pre-existing.	Diet: a personalized plan with recommendations on food choice, portion size, frequency of eating and pattern of intake Physical activity advice: Exercise 3–5 times per week (30–45 min per session) Among recommended were: walking, swimming, mild aerobics, stretching and strength exercise. Additionally participants received exercise instruction (VHS/DVD) to facilitate home-based exercise	Standard prenatal care recommended by the Society of Obstetricians and Gynaecologists of Canada24 Package of up-to-date information on physical activity and nutrition healthy pregnancy from the Health Canada	**GDM** (Definition: Canadian Diabetes Association’s clinical practice guidelines).
		**Allocation Concealment:** Sealed envelopes	**Exclusion Criteria:** Any medical, obstetric, skeletal or muscular disorders that could prevent women from taking part in physical exercise			excessive gestational weight gain (EGWG)
		**Blinding:** Participants and study staff were not blinded to the intervention				birth weight, macrosomia
**Jeffries 2009** ^**24**^	**286**	**Methods of Randomization:** Computer generated	**Inclusion Criteria:** Before 14 weeks of gestation	Given an optimal gestational weight gain range	Standard antenatal care	**GDM** (Definition: Not reported).
		**Allocation Concealment:** Opaque, sequentially numbered envelopes.	**Exclusion Criteria:**<18 years or >45 years, Type I or II diabetes, multiple pregnancies			preterm delivery, gestational age at delivery, pre-eclampsia, gestational hypertension, cesarean section
		**Blinding:** Participants were blinded to the purpose of the study				birth weight, shoulder dystocia
**Petrella 2013** ^**27**^	**66**	**Methods of Randomization:** A computer-generated random allocation in blocks of three	**Inclusion Criteria:** Pre-pregnancy BMI≥25 kg/m^2^, age 418 years and single pregnancy.	The Therapeutic Lifestyle Changes group diet: 1500 kcal/day; three main meals and three snacks. In case of increased physical activity program, the dietitian added an amount of 200 kcal/day for obese or 300 kcal/day for overweight women.	A simple nutritional booklet about for a healthy diet during pregnancy lifestyle, in agreement with Italian Guidelines.	**GDM** (Definition: ADA criteria)
		**Allocation Concealment:** Sealed in numbered white envelopes	**Exclusion Criteria:** Twin pregnancy, chronic diseases (i.e. diabetes mellitus, chronic hypertension, untreated thyroid diseases), GDM in previous pregnancies, smoking during pregnancy, previous bariatric surgery, women who just engaged in regular physical activity, dietary supplements or herbal products known to affect body weight, other medical conditions that might affect body weight, and plans to deliver outside our Birth Center			GWG, gestational age at delivery, Preterm Delivery. Gestational hypertension. Caesarean Section. Induction of labour.
		**Blinding:** Both gynecologist and dietitian knew the allocation of the patient				birth weight, admission to Neonatal Intensive Care Unit
**Phelan 2011** ^**28**^	**401**	**Methods of Randomization:** Randomization was computer-generated	**Inclusion Criteria:** Gestational age 10–16 weeks, BMI 19.8–40 kg/m^2^, nonsmoking, adults (aged.18 y), access to a telephone, and a singleton pregnancy	Standard care and a behavioral lifestyle intervention designed to prevent excessive weight gains during pregnancy. IOM guidelines for nutrition and weight during pregnancy and was designed with an eventual dissemination in mind.	Standard nutrition counseling	**GDM** (Definition: Not reported)
		**Allocation Concealment:** Opaque envelopes	**Exclusion Criteria:** Major health or psychiatric diseases, weight loss during pregnancy, or a history of 3 miscarriages			Excessive Gestational Weight Gain (EGWG), gestational hypertension/ pre-eclampsia, cesarean section, preterm delivery, gestational age at delivery, birth weight
		**Blinding:** Clinic staff and physicians were blinded to subject randomization				
**Polley 2002** ^**29**^	**120**	**Methods of Randomization:** Not reported	**Inclusion criteria:** Pregnant women, <20 weeks gestation,	Information (written and oral) in the following areas: (a) appropriate weight gain during pregnancy; (b) exercise during pregnancy; and (c) healthful eating during pregnancy	Standard prenatal care (standard nutrition counselling which emphasized a well-balanced dietary intake and advice to take a multivitamin Iron supplement)	**GDM** (Definition: Not reported)
		**Allocation Concealment:** Not reported	**Exclusion criteria:** Women younger than 18 years, underweight (BMI <19.8 kg/m^2^), >12 weeks gestation, high risk pregnancy (drug abuse, chronic health problems, previous complication during pregnancy or current multiple gestation)			Excessive Gestational Weight Gain (EGWG) Gestation age at delivery, preterm delivery (<36 weeks), cesarean delivery, pre-eclampsia, gestational hypertension
		**Blinding**: Not reported				birth weight, macrosomia (>4000 g),
**Poston 2013** ^**30**^ (pilot study)	**183**	**Methods of Randomization:** Web based	**Inclusion Criteria:** BMI > 30kg/m^2^, singleton pregnancy, gestational age >15 weeks and <17 weeks gestation.	Dietary advice: increased consumption of low GI foods; replacement of sugar sweetened beverages with low GI alternatives; reduction of intake of saturated fats by their replacement with monounsaturated and polyunsaturated fats. Physical activity advice: walking at a moderate intensity level	Routine antenatal care (diet and physical activity advice in accordance with local policies, based on NICE guidelines (UK))	**GDM** (Definition: Diagnosis was confirmed by fasting glucose > 5.1 mmol/L and or 1hr glucose > 10 mmol/L and or 2 hr glucose > 8.5 mmol/L according the International Association of the Diabetes and Pregnancy Study Groups guidelines.
		**Allocation Concealment:** Treatment allocation was done automatically using web based tool	**Exclusion criteria:** Gestation age <15 or >17,6 weeks; pre-existing diabetes; pre-existing essential hypertension (treated); pre-existing renal disease; multiple pregnancy; systemic lupus erythematosus (SLE); anti-phospholipid syndrome; sickle cell disease; thalassemia; celiac disease; currently prescribed metformin; thyroid disease or current psychosis.			GWG, pre-eclampsia, mode of delivery
		**Blinding:** Not reported				large for gestational age (LGA)
**Renault 2013** ^**32**^	**283**	**Methods of Randomization:** Web based randomization	**Inclusion Criteria:** Pre-pregnancy BMI ≥30 kg/m^2^, Before 16 weeks of gestation. Age older than 18 years. Singleton pregnancy. Normal scan at 11–14 weeks. Read and speak Danish	The dietary intervention: consultation with dietitian every 2 weeks, (outpatient visits and phone contacts) Physical activity monitored using validated pedometer counting the daily numbers of steps	Usual hospital standard regimen for obese pregnant women	**GDM** (Definition: 75g glucose 2 hs)
		**Allocation Concealment:** Web based allocation run by a third party	**Exclusion Criteria:** Multiple pregnancy, diabetes, any other serious diseases limiting physical activity, bariatric surgery, Alcohol or drugs abuse.			GWG, gestational hypertension, pre-eclampsia, induction of labour, caesarean section, gestational age at delivery, preterm delivery
		**Blinding:** Not reported				birth weight
**Vesco 2013** ^**34**^ (Conference Abstract)	**114**	**Methods of Randomization:** Using a computerized algorithm to generate the random assignments	**Inclusion Criteria:** Women with singleton gestation and a pre-pregnancy BMI ≥30 kg/m^2^	A weekly, group-based, weight management intervention designed to help limit GWG to 3% of weight (measured at the time of randomization)	Usual care	**GDM** (Definition: ADA criteria)
		**Allocation Concealment:** Not Reported	**Exclusion Criteria:** Gestational age >20 weeks, multiple pregnancy, anticipated disenrollment from KPNW prior to delivery, type 1 and type 2 diabetes mellitus (or a diagnosis of GDM prior to randomisation), other medical conditions requiring specialized medical care or conditions potentially affecting weight gain			GWG, gestational age at delivery, delivery type, cesarean section, preterm delivery, gestational hypertension/ pre-eclampsia
		**Blinding:** Clinical staff members were blinded to group assignment				birth weight, large for gestational age (LGA), small for gestational age (SGA)
**Vinter 2011** ^**35**^	**360**	**Methods of Randomization:** Computer-generated table of numbers	**Inclusion Criteria**: Pre- or early pregnancy BMI of 30–45 kg/m^2^, age 18–40 years, 10–14 weeks of gestation	Dietary counselling following official Danish recommendations aiming to limit GWG to 5 kg Moderately physically active (30–60 min daily) monitored using a pedometer. Indoor training consisting of aerobic with light weights and elastic bands, and balance exercises	Access advice about dietary habits and physical activities in pregnancy without any additional intervention	**GDM** (Definition: 2-h oral glucose tolerance test capillary blood glucose result was > or = 9 mmol/L).
		**Allocation Concealment:** Closed envelopes	**Exclusion Criteria**: Prior serious obstetrics complications, chronic diseases (hypertension, diabetes), positive OGTT in early pregnancy, Alcohol or drugs abuse, multiple pregnancy			GWG, pre-eclampsia, gestational hypertension. cesarean section,
		**Blinding:** Not reported				macrosomia, large for gestational age (LGA), admission to Neonatal Intensive Care Unit.

GDM—gestational diabetes mellitus; GWG—Gestational Weight Gain; ADIPS—the Australian Diabetes in Pregnancy Society; ADA—according to the American Diabetes Association;

### Quality of the included studies

All studies with diet based interventions had low risk of bias for adequate randomisation and attrition [25,26,31,33,36,37] ; half of them (3/6) had low risk for allocation concealment [26,31,36] . Only one study reported adequate blinding of researchers and participants [26] and 33% were considered as high risk in this domain (2/6) [25,36] . The blinding of outcomes assessors was adequate in one diet study [31] and inadequate in one [25] . The risk of bias due to selective reporting was low in 33% of the included diet based studies (2/6) [26,31] and inadequate in other two trials [25,36] .

Twelve trials on mixed (diet and lifestyle) approach had a low risk of bias for adequate randomisation (92%, 12/13). [18,20–24,27,28,30,32,34,35] Allocation concealment was well described in six [20,22,27,28,30,32] out of 13 mixed approach interventions studies. Blinding of staff and participants was adequate in one trial [22] and inadequate in two; and blinding of outcomes assessment was correct in 31% of included trials (4/13) [20,22,28,34]. Attrition bias was low for majority of studies except one study that was at high risk [23]. Selective reporting bias was low in 54% of mixed approach trials [18,20,22–24,28,29] and was considered to be high in four trials (31%) [21,27,32,35].

**Fig 2:**
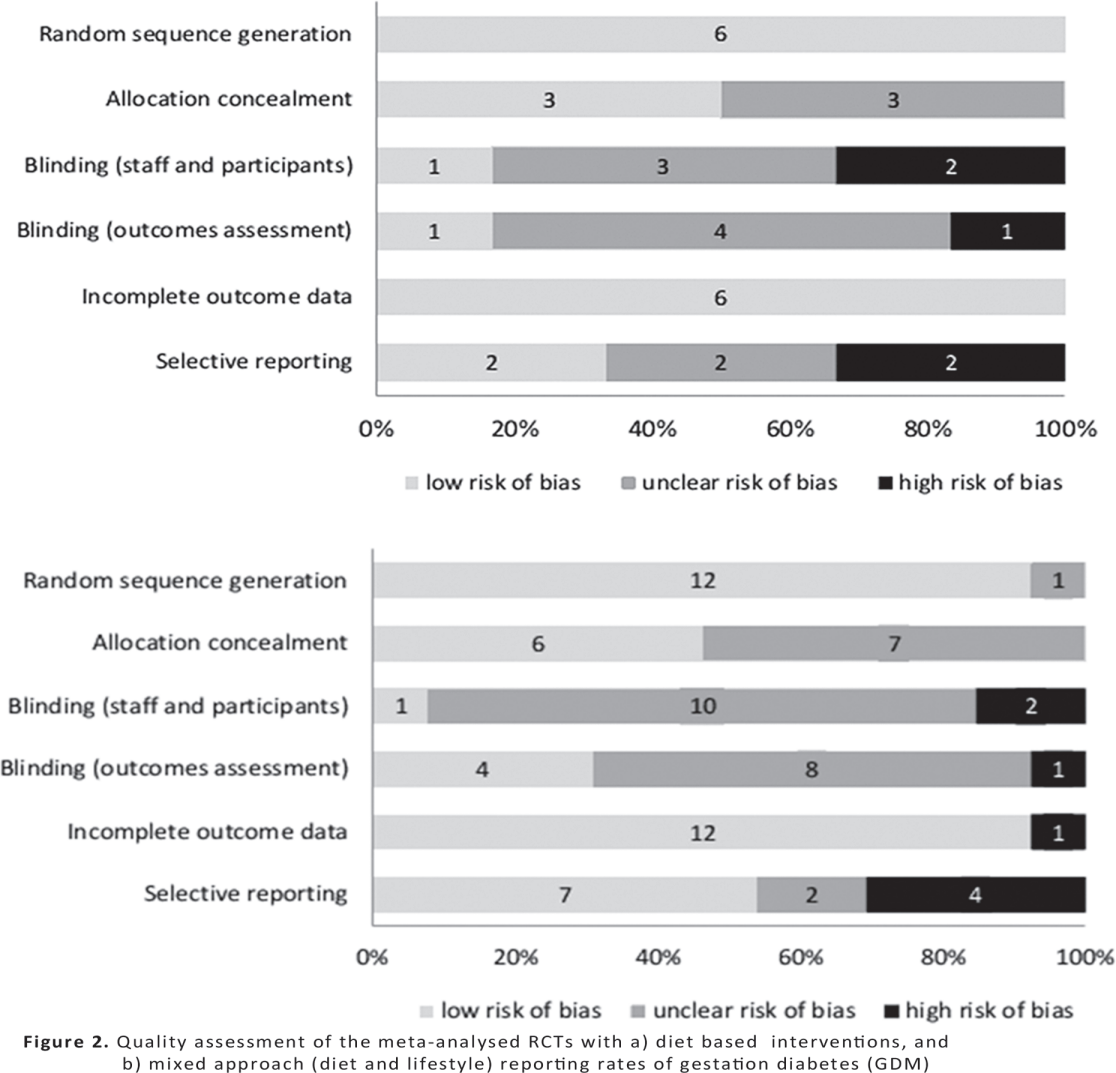
Quality assessment of the meta-analysed RCTs with a) diet based interventions, and b) mixed approach (diet and lifestyle) reporting rates of gestation diabetes (GDM).

Of the two trials on nutritional supplements, the trial on *myo*-inositol supplementation [19] was assessed to have low risk of bias for adequate randomisation, attrition and selective reporting and high risk for blinding of staff and participants. The trial on probiotics had low risk of bias for all domains except for detection bias. [26] Fig. 2 provides the quality assessment of the included studies for diet based and mixed approach groups.

### Effect of nutritional manipulation on GDM

**Fig 3:**
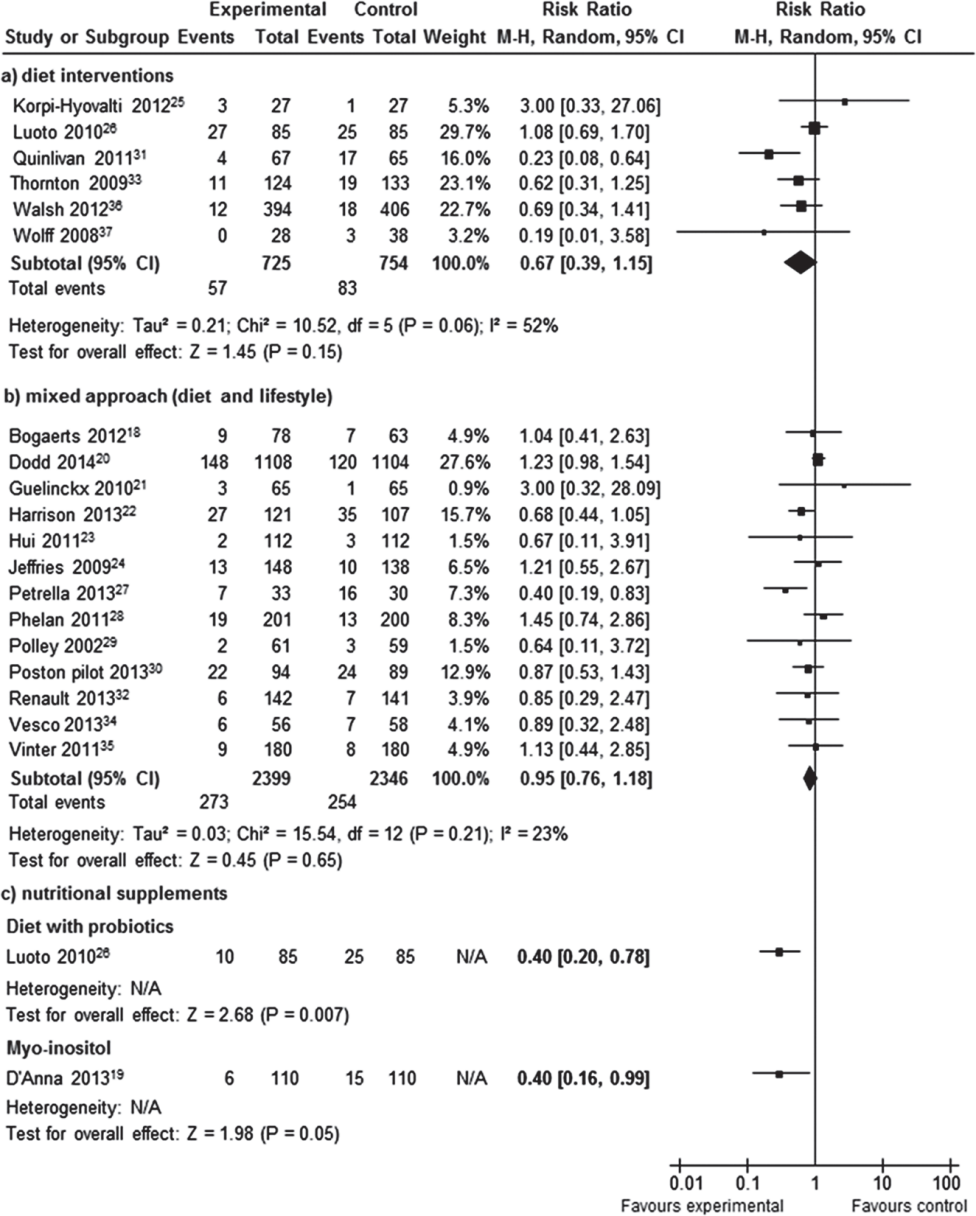
Forest plot of the meta-analysed RCTs with a) diet interventions, b) mixed approach (diet and lifestyle), and c) nutritional supplements reporting rates of gestation diabetes (GDM).

Interventions that were mainly based on diet reduced the rates of GDM by 33% (RR 0.67; 95% CI 0.39, 1.15I^2^ = 52%) (Fig. 3a). There were no differences between the two groups for mixed (diet and lifestyle) approach (RR 0.95; 95% CI 0.89, 1.22; I^2^ = 23%) (Fig. 3b). The risk of GDM was reduced by 60% for probiotics (with diet) in comparison to standard care (RR 0.40; 95% CI 0.20, 0.78; p<0.01). A similar reduction was observed for the nutritional supplement *myo*-inositol (RR 0.40; 95% CI 0.16, 0.99; p = 0.05) (Fig. 3c).

### Small study effect

Due to insufficient number of studies for *myo*-inositol and diet with probiotic supplementation we examined funnel plot asymmetry only for diet based and mixed approach groups. For both groups Harbord’s statistical tests for small study effect was insignificant (p = 0.87 and p = 0.21, respectively),

### Subgroup analysis

Subgroup comparison was possible to conduct only for two intervention categories: diet based and mixed approach. There was no subgroup differences based on maternal risk for GDM for both intervention groups and for BMI category comparison for the mixed approach group (Table 2). There was a significant difference according to the BMI for diet based intervention (p = 0.04). A significant reduction in GDM was observed in the subgroup comprised of obese and overweight women (RR 0.40; 95% CI 0.18, 0.86, I^2^ = 29%).

**Table 2:** Subgroup analyses for intervention types and clinical characteristics for gestational diabetes mellitus (GDM) in evaluation of nutritional manipulation in pregnancy.

	Gestational diabetes mellitus		
Subgroup	No of studies	Risk Ratio (95% CI)	P value for interaction
**Diet based interventions**			
**Risk status**			
High risk	5^25,31,33,36,37^	0.55 (0.30, 1.00)	0.08
Low risk	1^26^	0.40 (0.20, 7.80)	
**BMI category**			
Obese and overweight	3^31,33,37^	0.40 (0.18, 0.86)	**0.04**
Any weight	3^25, 26,36^	0.98 (0.65, 1.47)	
**Mixed approach (diet and lifestyle)**			
**Risk status**			
High risk	7^18,21,22,30,32,34,35^	0.83 (0.64, 1.09)	0.19
Low risk	6^20,23,24,27–29^	0.97 (0.64, 1.48)	
**BMI category**			
Obese and overweight	9^18,20–22,27,30,32,34,35^	1.02 (0.86, 1.20)	0.44
Any weight	4^23,24,28,29^	1.20 (0.75, 1.93)	

### Effect of nutritional interventions on other maternal and neonatal outcomes

#### Maternal outcomes

Eleven trials [19,20,22,24,27–29,32–34,36] evaluated the role of interventions in preventing preterm delivery before 37 weeks of gestation None of the interventions significantly reduced the rate of preterm deliveries; however, the risk reduction was the highest (51%) for diet based group (RR 0.49, 95% CI 0.19, 1.29).

Fourteen RCTs [18–20,23,24,26–29,32–36] reported the effect of interventions on the caesarean section rate and six studies [18,20,27,32,33,36] on the rate of the induction of labour. There was no significant effect of the intervention on the rates of evaluated outcomes (Table 3).

**Table 3:** Summary of findings for maternal and neonatal outcomes from trials with nutritional manipulation in pregnancy.

Outcome	Intervention	No of studies	Sample size	Risk Ratio* (95% CI)	I^2^	*P value*
**Maternal outcomes**						
**Preterm delivery (< 37weeks)**	Diet based interventions	2^33,36^	1,057	0.49 (0.19, 1.29)	0%	0.15
	Mixed approach	8^20,22,24,27–29,32,34^	3,697	0.84 (0.55, 1.27)	27%	0.40
	Myo-inositol	1^19^	220	0.75 (0.17, 3.27)	N/A	0.70
**Caesarean section**	Diet based interventions	3^26,33,37^	494	1.17 (0.99, 1.38)	0%	0.06
	Mixed approach	10^18,20,23,24,27–29,32,34,35^	4,194	0.91 (0.82, 1.02)	7%	0.10
	Myo-inositol	1^19^	220	0.98 (0.70, 1.36)	N/A	0.89
	Diet with probiotics	1^26^	170	1.09 (0.51, 2.33)	N/A	0.82
**Induction of labour**	Diet based interventions	2^33,36^	1,057	1.14 (0.54, 2.40)	83%	0.74
	Mixed approach	4^18,20,27,32^	2,689	1.02 (0.91, 1.13)	0%	0.78
**Gestational hypertension**	Diet based interventions	2^33,37^	323	0.16 (0.02, 1.11)	19%	0.06
	Mixed approach	7^18,20,24,27,-29,32^	3,496	0.93 (0.68, 1.26)	17%	0.63
	Myo-inositol	1^19^	220	1.50 (0.26, 8.80)	N/A	0.65
**Pre-eclampsia**	Diet based interventions	2^33,36^	323	0.66 (0.27, 1.59)	0%	0.36
	Mixed approach	7^18,20,24,28,29,32,35^	3,793	0.96 (0.75, 1.24)	0%	0.77
**Fetal outcomes**						
**Birth weight**	Diet based interventions	5^25,31,33,36,37^	1,219	0.06^#^ (-0.13, 0.25)	46%	0.53
	Mixed approach	6^18,23,24,27,28,34^	1,088	0.04^#^ (-0.17, 0.24)	65%	0.73
	Myo-inositol	1^19^	220	-0.51^#^ (-0.79, -0.22)	N/A	**<0.01**
**Shoulder dystocia**	Diet based interventions	1^36^	800	0.52 (0.09, 2.80)	N/A	0.44
	Mixed approach	2^20,24^	2,506	1.24 (0.81, 1.91)	0%	0.33
	Myo-inositol	1^19^	220	0.50 (0.05, 5.43)	N/A	0.57
**Admission to NICU**	Mixed approach	2^20,35^	2,562	1.01 (0.91, 1.13)	0%	0.82
**Neonatal death**	Mixed approach	1^20^	2,212	3.99 (0.45, 35.60)	N/A	0.22
**Stillbirth**	Mixed approach	1^20^	2,212	1.00 (0.29, 3.43)	N/A	1.00

*Random effect model

^#^ SMD—standardized mean difference

Ten studies reported on the effects of the intervention on gestational hypertension [18–20,24,27–29,32,33,37] and nine on pre-eclampsia [18,20,24,28,29,32,33,35,37]. The risk of gestational hypertension and pre-eclampsia was reduced by 84% and 34%, respectively, in diet-based group (Table 3). There was no noticeable effect of mixed approach on the occurrence of discussed outcomes.

#### Fetal outcomes

Ten trials evaluated the effect of nutritional manipulation in pregnancy on the birth weight of the newborns. [18,19,23–25,27,28,31,33,34,36,37] The only significant difference in birth weight between the groups was recorded for *myo*-inositol (SMD-0.51, 95% CI-0.79, -0.22; p<0.01). Four trials [19,20,24,36] reported on shoulder dystocia, and showed no significant difference between the groups for any of three intervention groups (Table 3).

In two studies evaluating mixed approach (2,562 women) authors reported the rates of the admissions to Neonatal Intensive Care Unit. [20,35] There was no statistically significant difference in numbers of admissions between the intervention and the control group (RR 1.01, 95% CI 0.91, 1.13; I^2^ = 0%). Only one study [20] reported events of neonatal death and stillbirth. For both outcomes estimated risks were none significant (Table 3).

#### Adverse effects

Nine of the 20 trials reported or evaluated the adverse effects of the interventions on the mother and offspring. [19,23,26,28,29,31,33,36,37] No significant adverse effects were observed for *myo*-inositol or probiotics in studies that exposed women to the intervention in the first trimester. One study [31], assessed the impact of the diet based intervention and reported a case of severe intrauterine growth restriction in each of the arms that resulted in preterm delivery.

## Discussion

### Summary of findings

Nutritional manipulation based on diet or mixed approach does not appear to prevent GDM. There was a trend towards beneficial effect in women on mainly diet-based intervention, with a potential for significant reduction in GDM risk when limited to obese and overweight women. Nutritional supplements such as probiotics and *myo*-inositol show promising role in the strategy for primary prevention of GDM.

### Relevance to current evidence

Until now, there has been no robust evidence to provide guidance on the primary prevention of GDM due to the small number of studies limited to few interventions in published reviews. [38] The number of eligible studies has doubled since our previous review that evaluated the effect of mixed approach (diet and lifestyle modification) on GDM. [11] By evaluating all the relevant interventions, our review is the first to systematically assess the effects of nutritional manipulation in pregnancy on GDM. We complied with current guidelines and used a comprehensive search strategy without any language restrictions. By including only randomised trials, we avoided some of the pitfalls encountered by earlier reviews that included quasi-randomised studies [10] and women with GDM [9].

### Effects of interventions on GDM

Amongst evaluated interventions, diet based interventions appear to show potential for preventing GDM. This could be due to the following reasons: individual dietary and components; change in gestational weight gain and effect of nutritional supplements.

The interventions promoted the uptake of healthy components such as fibre, probiotics and food rich in vitamins such as *myo*-inositol that may have an additive effect in reducing the concentrations of maternal glucose. [19,26] The women in the intervention group had reduced total energy intake and glycaemic load compared to the controlled group. [30,36] Low glycaemic index diet attenuates the increase in insulin resistance observed in pregnancy, thereby reducing the risk of GDM. [39] The risk of GDM is known to be reduced by a quarter with each 10-g/day increment in total fibre intake. [40] The largest benefit with diet was observed where there was a multidisciplinary input into the intervention, with the use of food diaries [31] and feedback methods.

Diet based interventions have also shown the greatest reduction in gestational weight gain compared to other methods. [11] The reduction in gestational weight gain may have influenced the fall in the rates of GDM. [11] Serum leptin, a known factor associated with GDM [41], was lowered by 20% with reduced gestational weight gain. [37] Cord leptin concentrations were also increased in newborns born to mothers with diabetes. [42]

We did not observe the beneficial effect in the subgroup with mixed approach that combined diet and physical activity. This is consistent with previously published reviews that did not show beneficial effect of physical activity in pregnancy on pregnancy outcomes. [11,43] Rather than physical activity failing to have an expected impact on GDM, it is likely that women in the intervention group had poor compliance with the intervention. Objective assessments with methods such as accelerometry have shown no difference in the physical activity between the two groups. [30] The largest trial on mixed approach (diet and lifestyle) in pregnancy, the LIMIT study failed to show a benefit with the intervention for GDM and other maternal outcomes including gestational weight gain.[20] Non-compliance with the intervention, with a quarter of women not attending the required two sessions with the dietician could have contributed to the lack of benefit.

Simple interventions based on nutritional supplements such as *myo*-inositol and probiotics appear to have significant potential in preventing GDM. Inositol is available in cereals, meat, fresh fruit and vegetables, corn and legumes. The average dietary intake contains 1g of inositol/day. Myo-inositol is known to increase the sensitivity to insulin, [44] a possible mechanism for the observed reduction in GDM.

Other supplements such as the probiotics, consisting of microorganisms of beneficial nature, appear to reduce the risk of GDM when combined with a dietary intervention. [26] By altering the gut microbiome, and by modifying the concentration of plasma lipopolysaccharides, probiotics alter the inflammatory pathways and sensitivity to insulin. It is possible that the benefit observed in the Luoto trial in reducing GDM [26] was due to a synergistic action between a diet rich in probiotics in addition to probiotics supplements.

### Safety of the interventions

Any intervention evaluated in pregnancy needs to pass a rigorous evaluation of its safety to the mother and baby. Our previous detailed evaluation of diet and mixed interventions in pregnancy did not find adverse effects to the mother or baby, except in extreme conditions such as starvation. [11] Although theoretical concerns have been raised regarding the risk of preterm delivery with inositol, this was not observed in both randomised and observational studies on inositol in pregnancy. Inositol use in early pregnancy may in an additional beneficial role, by preventing the risk of neural tube defect in folate resistant mothers. [45]

### Limitations

Our findings were limited by differences in the inclusion criteria of the studies, variation in the components of the intervention such as duration, intensity and frequency, non-standardised care in the control group and non-uniform definitions of GDM. Furthermore, women in the intervention group had more than one intervention, such as diet and probiotics, making it difficult to delineate the beneficial effect of an individual intervention. It is possible that a different criterion for the diagnosis of gestational diabetes may have yielded changed estimates of effect. [46] Women in the control group may have accessed these interventions resulting in Hawthorne effect for the following reasons: interventions were easily accessible, including over the counter nutritional supplement; and absence of blinding of the women or health care provider, in any of the included studies. None of the studies evaluated GDM as a primary outcome. Hence it is possible that the different arms could have been treated differently, such as additional screening for GDM, and close follow-up in the intervention group, thereby influencing the outcome. Studies were limited in their reporting on proportion of women who complied with the intervention, which could have a major influence on the effect size observed.

### Clinical applicability

Since women with GDM are mostly seen in the secondary care, with frequent follow ups including ultrasound assessment of fetal growth, any effective intervention that prevents GDM is likely to be cost effective in the long run. Dietary interventions are complex, and require a change in the behaviour of mothers, to have a positive impact on the outcomes. Furthermore, they require reinforcement and feedback with food diaries, and regular visits with healthcare professionals such as dieticians, midwives and clinicians. The diet based intervention may have a role in primary prevention of GDM, especially in obese and overweight pregnant women.

With a projected increase in the National Health Service (NHS) spend from £8.8 billion to £13 billion per year in the next 25 years on Type 2 diabetes and its complications, [47] primary prevention of GDM has significant societal and economic benefits. Interventions based on diet and nutritional supplements show potential to prevent GDM, with the possibility of promoting the health of subsequent generations, by reducing the risks of obesity and adult onset diabetes in children born to mothers with GDM.

### Research recommendations

The role of diet-based interventions in obese and overweight pregnant women, the population most likely to develop GDM, needs further evaluation. The beneficial effects of simple interventions such as probiotics and *myo*-inositol on GDM appear promising. There is a need to evaluate the effects of supplements by large multicentre randomised trials, involving wider group of individuals such as non-Caucasians and obese women. The optimal dose, frequency and type of inositol isomer need to be identified. Similarly the effects of different genera or strains of probiotics and their varied dose on GDM need to be identified. Given the considerable resources required to deliver the complex interventions based on diet, it is possible that nutritional supplements will also be cost effective. Furthermore, they are an attractive option as they are easily available as over the counter supplements.

## Conclusion

Mixed approach interventions composed of diet and lifestyle modification do not appear to prevent GDM. Diet based interventions may be beneficial in obese and overweight pregnant women. Nutritional supplements such as probiotics and *myo*-inositol show benefit and need further evaluation in large randomised trials.

## Supporting Information

S1 Appendix Search strategies for MEDLINE via Ovid. (DOCX)

S2 Appendix Quality assessment of included studies. (DOCX)

S3 Appendix Completed PRISMA Checklist. (DOC)
