# Supplementary material for: Nutritional Manipulation for the Primary Prevention of Gestational Diabetes Mellitus: A Meta-Analysis of Randomised Studies
Source: PLoS One. 2015 Feb 26;10(2):e0115526. doi: 10.1371/journal.pone.0115526 (PMC4342242; doi:10.1371/journal.pone.0115526)
Supplement: S1 Appendix — (DOCX) [file pone.0115526.s001.docx]

**Appendix S1** Search strategies used in MEDLINE (via Ovid) the systematic review on nutritional manipulation in the prevention of gestational diabetes

a) Diet based and mixed interventions

1 Gestational Diabetes Mellitus/

2 exp Gestational Diabetes Mellitus/

3 Gestation* Diabetes.mp.

4 GDM.mp.

5 maternal diabetes.mp.

6 (pregnancy induced adj3 diabetes).tw.

7 1 or 2 or 3 or 4 or 5 or 6

8 diet/

9 exp diet/

10 diet?.mp.

11 nutrition.mp.

12 8 or 9 or 10 or 11

13 7 and 12

14 Humans/

15 13 and 14

b) Myo-inositol

1 Gestational Diabetes Mellitus/

2 exp Gestational Diabetes Mellitus/

3 Gestation* Diabetes.mp.

4 GDM.mp.

5 maternal diabetes.mp.

6 (pregnancy induced adj3 diabetes).tw.

7 1 or 2 or 3 or 4 or 5 or 6

8 inositol/

9 exp inositol/

10 Myoinositol/

11 exp Myoinositol/

12 Myo-inositol.mp.

13 8 or 9 or 10 or 11 or 12

14 7 and 13

c) Probiotics

1 Gestational Diabetes Mellitus/

2 exp Gestational Diabetes Mellitus/

3 Gestation* Diabetes.mp.

4 GDM.mp.

5 maternal diabetes.mp.

6 (pregnancy induced adj3 diabetes).tw.

7 1 or 2 or 3 or 4 or 5 or 6

8 exp prebiotics/ or exp probiotics/

9 exp Lactobacillus/

10 lactobacillus.mp.

11 bifidobacterium.mp.

12 exp Bifidobacterium/

13 8 or 9 or 10 or 11 or 12

14 yakult.mp.

15 actimel.mp.

16 yoghurt.mp.

17 Kefir.mp.

18 Kimchi.mp.

19 kombucha.mp.

20 probiotic?.mp.

21 14 or 15 or 16 or 17 or 18 or 19 or 20

22 13 or 21

23 7 and 22

d) Vitamin D

1 Gestational Diabetes Mellitus/

2 exp Gestational Diabetes Mellitus/

3 Gestation* Diabetes.mp.

4 GDM.mp.

5 maternal diabetes.mp.

6 (pregnancy induced adj3 diabetes).tw.

7 1 or 2 or 3 or 4 or 5 or 6

8 Vitamin D/

9 exp Vitamin D/

10 Cholecalciferol/

11 exp Cholecalciferol/

12 8 or 9 or 10 or 11

13 7 and 12
