# Supplementary material for: Nutritional Manipulation for the Primary Prevention of Gestational Diabetes Mellitus: A Meta-Analysis of Randomised Studies
Source: PLoS One. 2015 Feb 26;10(2):e0115526. doi: 10.1371/journal.pone.0115526 (PMC4342242; doi:10.1371/journal.pone.0115526)
Supplement: S2 Appendix — (DOCX) [file pone.0115526.s002.docx]

**Appendix S2** Quality of individual studies included in the systematic review on nutritional manipulation in the prevention of gestational diabetes (+ low risk of bias;? unclear risk of bias; - high risk of bias)
